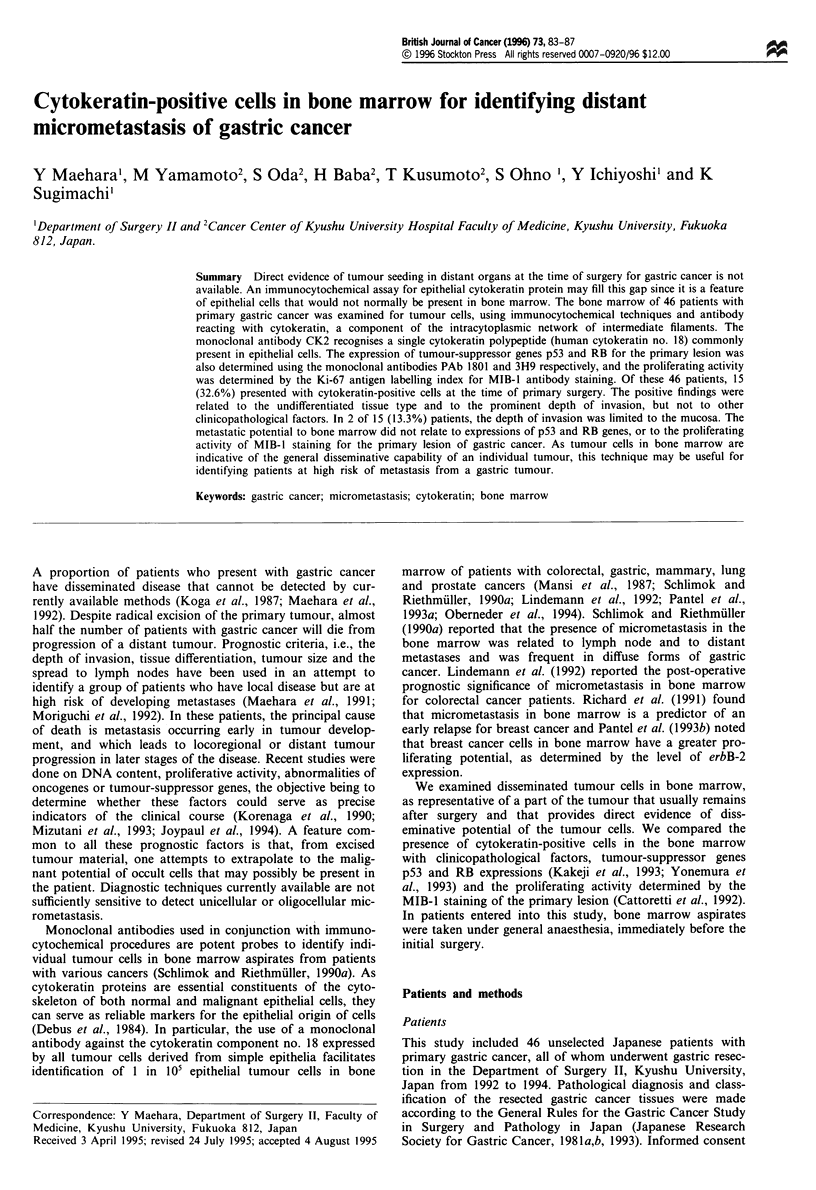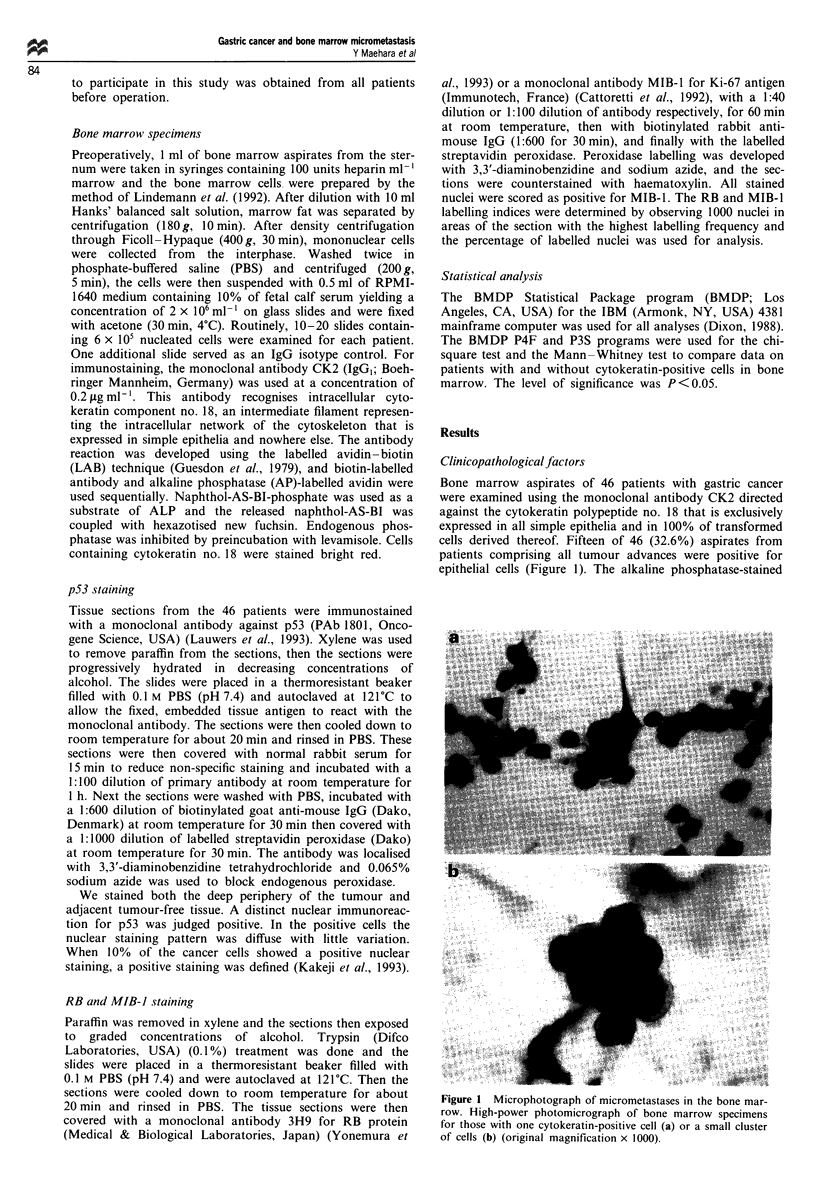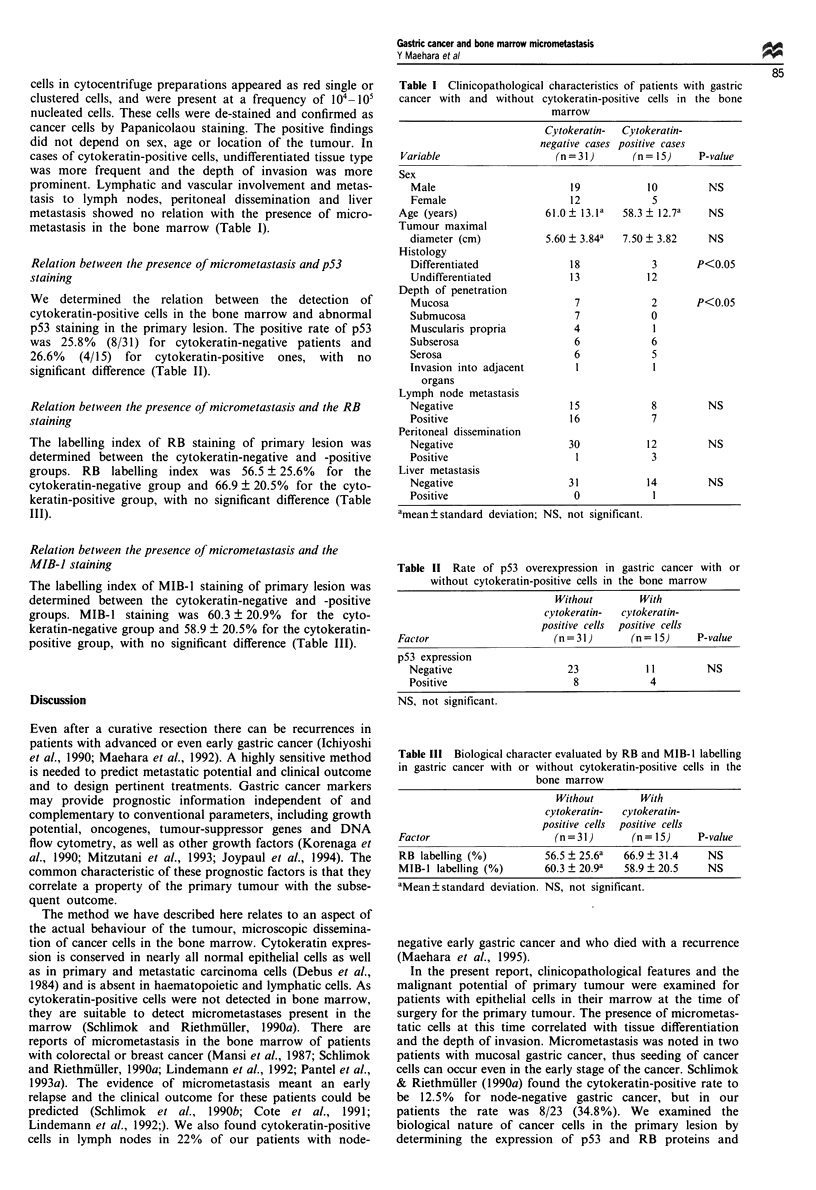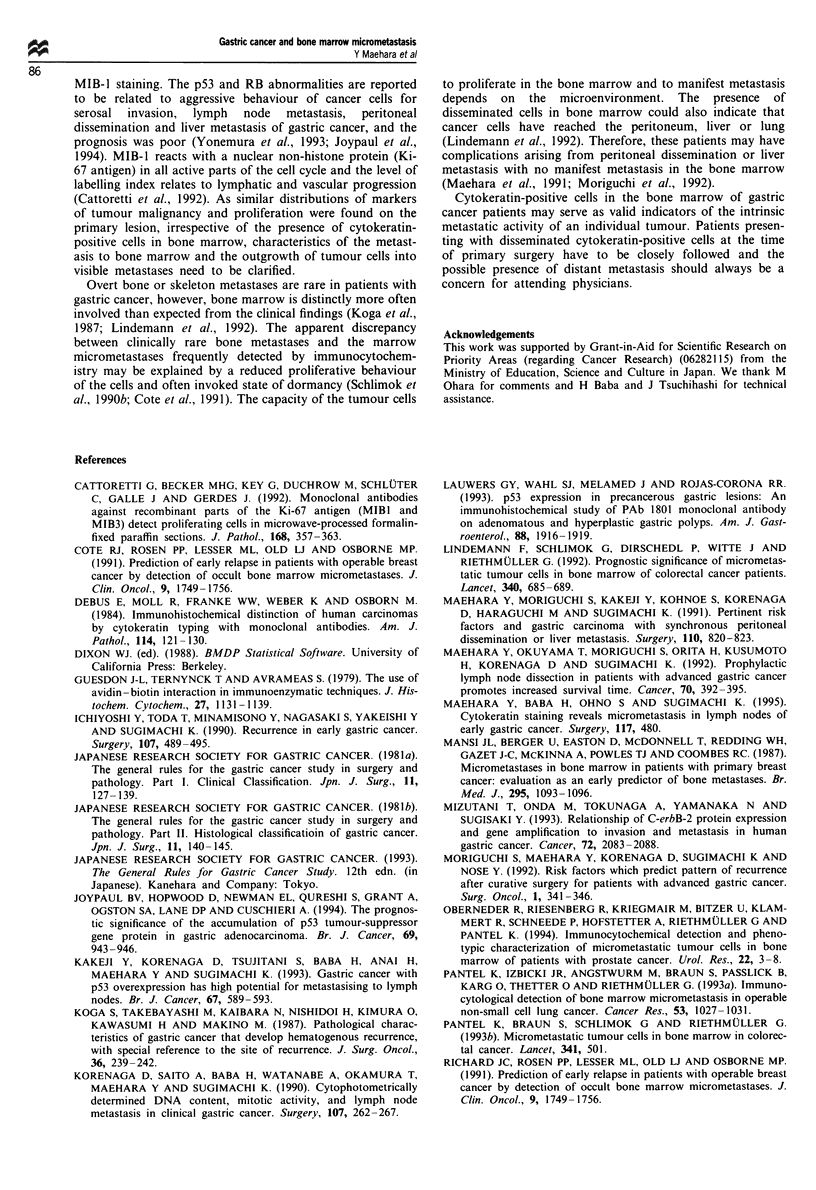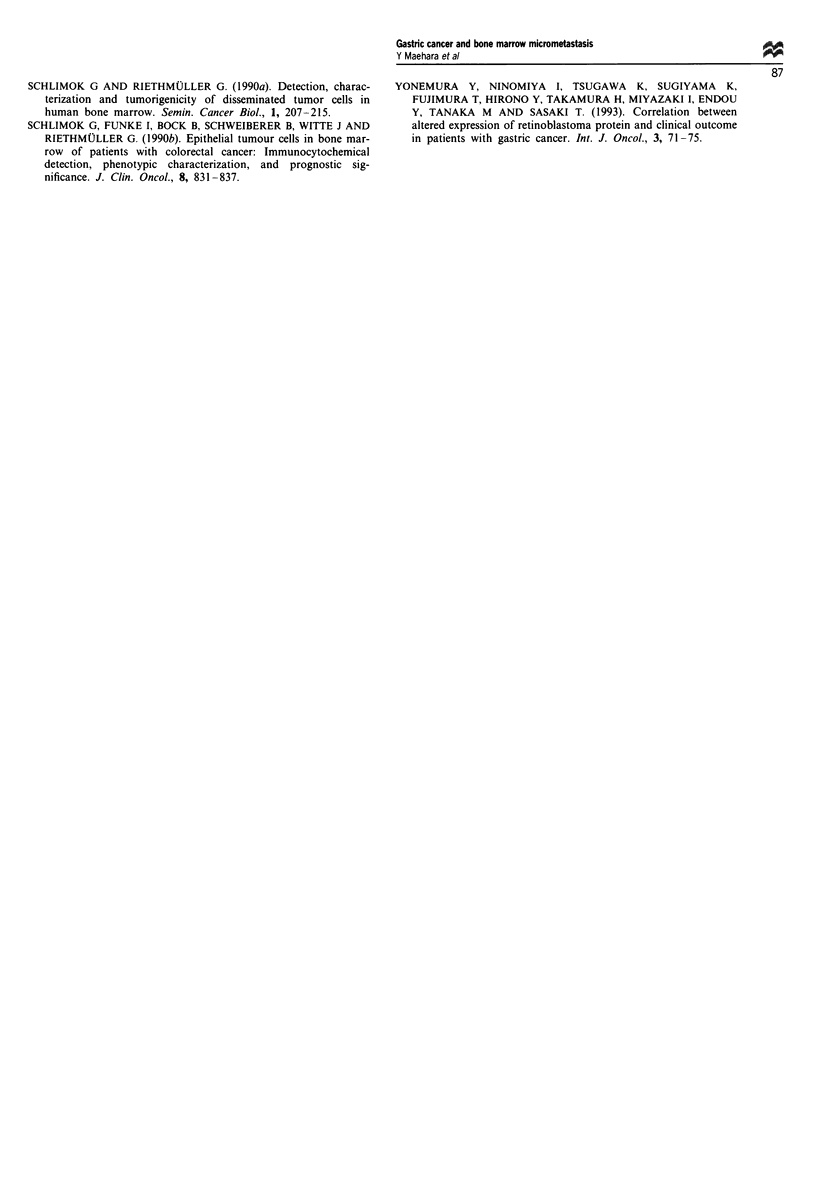# Cytokeratin-positive cells in bone marrow for identifying distant micrometastasis of gastric cancer.

**DOI:** 10.1038/bjc.1996.15

**Published:** 1996-01

**Authors:** Y. Maehara, M. Yamamoto, S. Oda, H. Baba, T. Kusumoto, S. Ohno, Y. Ichiyoshi, K. Sugimachi

**Affiliations:** Department of Surgery II, Kyushu University Hospital Faculty of Medicine, Kyushu University, Fukuoka, Japan.

## Abstract

**Images:**


British Journal of Cancer (1996) 73, 83-87
© 1996 Stockton Press All rights reserved 0007-0920/96 $12.00

### Cytokeratin-positive cells in bone marrow for identifying distant micrometastasis of gastric cancer

Y Maehara[1], M Yamamoto[2], S Oda[2], H Baba[2], T Kusumoto[2], S Ohno [1], Y Ichiyoshi[1] and K Sugimachi[1]

[1]*Department of Surgery II and* [2]*Cancer Center of Kyushu University Hospital Faculty of Medicine, Kyushu University, Fukuoka 812, Japan.*

**Summary** Direct evidence of tumour seeding in distant organs at the time of surgery for gastric cancer is not available. An immunocytochemical assay for epithelial cytokeratin protein may fill this gap since it is a feature of epithelial cells that would not normally be present in bone marrow. The bone marrow of 46 patients with primary gastric cancer was examined for tumour cells, using immunocytochemical techniques and antibody reacting with cytokeratin, a component of the intracytoplasmic network of intermediate filaments. The monoclonal antibody CK2 recognises a single cytokeratin polypeptide (human cytokeratin no. 18) commonly present in epithelial cells. The expression of tumour-suppressor genes p53 and RB for the primary lesion was also determined using the monoclonal antibodies PAb 1801 and 3H9 respectively, and the proliferating activity was determined by the Ki-67 antigen labelling index for MIB-1 antibody staining. Of these 46 patients, 15 (32.6%) presented with cytokeratin-positive cells at the time of primary surgery. The positive findings were related to the undifferentiated tissue type and to the prominent depth of invasion, but not to other clinicopathological factors. In 2 of 15 (13.3%) patients, the depth of invasion was limited to the mucosa. The metastatic potential to bone marrow did not relate to expressions of p53 and RB genes, or to the proliferating activity of MIB-1 staining for the primary lesion of gastric cancer. As tumour cells in bone marrow are indicative of the general disseminative capability of an individual tumour, this technique may be useful for identifying patients at high risk of metastasis from a gastric tumour.

**Keywords:** gastric cancer; micrometastasis; cytokeratin; bone marrow

A proportion of patients who present with gastric cancer have disseminated disease that cannot be detected by currently available methods (Koga *et al.*, 1987; Maehara *et al.*, 1992). Despite radical excision of the primary tumour, almost half the number of patients with gastric cancer will die from progression of a distant tumour. Prognostic criteria, i.e., the depth of invasion, tissue differentiation, tumour size and the spread to lymph nodes have been used in an attempt to identify a group of patients who have local disease but are at high risk of developing metastases (Maehara *et al.*, 1991; Moriguchi *et al.*, 1992). In these patients, the principal cause of death is metastasis occurring early in tumour development, and which leads to locoregional or distant tumour progression in later stages of the disease. Recent studies were done on DNA content, proliferative activity, abnormalities of oncogenes or tumour-suppressor genes, the objective being to determine whether these factors could serve as precise indicators of the clinical course (Korenaga *et al.*, 1990; Mizutani *et al.*, 1993; Joypaul *et al.*, 1994). A feature common to all these prognostic factors is that, from excised tumour material, one attempts to extrapolate to the malignant potential of occult cells that may possibly be present in the patient. Diagnostic techniques currently available are not sufficiently sensitive to detect unicellular or oligocellular micrometastasis.

Monoclonal antibodies used in conjunction with immunocytochemical procedures are potent probes to identify individual tumour cells in bone marrow aspirates from patients with various cancers (Schlimok and Riethmüller, 1990*a*). As cytokeratin proteins are essential constituents of the cytoskeleton of both normal and malignant epithelial cells, they can serve as reliable markers for the epithelial origin of cells (Debus *et al.*, 1984). In particular, the use of a monoclonal antibody against the cytokeratin component no. 18 expressed by all tumour cells derived from simple epithelia facilitates identification of 1 in $10^5$ epithelial tumour cells in bone marrow of patients with colorectal, gastric, mammary, lung and prostate cancers (Mansi *et al.*, 1987; Schlimok and Riethmüller, 1990*a*; Lindemann *et al.*, 1992; Pantel *et al.*, 1993*a*; Oberneder *et al.*, 1994). Schlimok and Riethmüller (1990*a*) reported that the presence of micrometastasis in the bone marrow was related to lymph node and to distant metastases and was frequent in diffuse forms of gastric cancer. Lindemann *et al.* (1992) reported the post-operative prognostic significance of micrometastasis in bone marrow for colorectal cancer patients. Richard *et al.* (1991) found that micrometastasis in bone marrow is a predictor of an early relapse for breast cancer and Pantel *et al.* (1993*b*) noted that breast cancer cells in bone marrow have a greater proliferating potential, as determined by the level of *erb*B-2 expression.

We examined disseminated tumour cells in bone marrow, as representative of a part of the tumour that usually remains after surgery and that provides direct evidence of disseminative potential of the tumour cells. We compared the presence of cytokeratin-positive cells in the bone marrow with clinicopathological factors, tumour-suppressor genes p53 and RB expressions (Kakeji *et al.*, 1993; Yonemura *et al.*, 1993) and the proliferating activity determined by the MIB-1 staining of the primary lesion (Cattoretti *et al.*, 1992). In patients entered into this study, bone marrow aspirates were taken under general anaesthesia, immediately before the initial surgery.

### Patients and methods

### *Patients*

This study included 46 unselected Japanese patients with primary gastric cancer, all of whom underwent gastric resection in the Department of Surgery II, Kyushu University, Japan from 1992 to 1994. Pathological diagnosis and classification of the resected gastric cancer tissues were made according to the General Rules for the Gastric Cancer Study in Surgery and Pathology in Japan (Japanese Research Society for Gastric Cancer, 1981*a*,*b*, 1993). Informed consent

Correspondence: Y Maehara, Department of Surgery II, Faculty of Medicine, Kyushu University, Fukuoka 812, Japan
Received 3 April 1995; revised 24 July 1995; accepted 4 August 1995

to participate in this study was obtained from all patients before operation.

*Bone marrow specimens*

Preoperatively, 1 ml of bone marrow aspirates from the sternum were taken in syringes containing 100 units heparin $ml^{-1}$ marrow and the bone marrow cells were prepared by the method of Lindemann *et al.* (1992). After dilution with 10 ml Hanks' balanced salt solution, marrow fat was separated by centrifugation (180 **g**, 10 min). After density centrifugation through Ficoll–Hypaque (400 **g**, 30 min), mononuclear cells were collected from the interphase. Washed twice in phosphate-buffered saline (PBS) and centrifuged (200 **g**, 5 min), the cells were then suspended with 0.5 ml of RPMI-1640 medium containing 10% of fetal calf serum yielding a concentration of $2 \times 10^6$ $ml^{-1}$ on glass slides and were fixed with acetone (30 min, 4°C). Routinely, 10–20 slides containing $6 \times 10^5$ nucleated cells were examined for each patient. One additional slide served as an IgG isotype control. For immunostaining, the monoclonal antibody CK2 ($IgG_1$; Boehringer Mannheim, Germany) was used at a concentration of 0.2 $\mu g$ $ml^{-1}$. This antibody recognises intracellular cytokeratin component no. 18, an intermediate filament representing the intracellular network of the cytoskeleton that is expressed in simple epithelia and nowhere else. The antibody reaction was developed using the labelled avidin–biotin (LAB) technique (Guesdon *et al.*, 1979), and biotin-labelled antibody and alkaline phosphatase (AP)-labelled avidin were used sequentially. Naphthol-AS-BI-phosphate was used as a substrate of ALP and the released naphthol-AS-BI was coupled with hexazotised new fuchsin. Endogenous phosphatase was inhibited by preincubation with levamisole. Cells containing cytokeratin no. 18 were stained bright red.

*p53 staining*

Tissue sections from the 46 patients were immunostained with a monoclonal antibody against p53 (PAb 1801, Oncogene Science, USA) (Lauwers *et al.*, 1993). Xylene was used to remove paraffin from the sections, then the sections were progressively hydrated in decreasing concentrations of alcohol. The slides were placed in a thermoresistant beaker filled with 0.1 M PBS (pH 7.4) and autoclaved at 121°C to allow the fixed, embedded tissue antigen to react with the monoclonal antibody. The sections were then cooled down to room temperature for about 20 min and rinsed in PBS. These sections were then covered with normal rabbit serum for 15 min to reduce non-specific staining and incubated with a 1:100 dilution of primary antibody at room temperature for 1 h. Next the sections were washed with PBS, incubated with a 1:600 dilution of biotinylated goat anti-mouse IgG (Dako, Denmark) at room temperature for 30 min then covered with a 1:1000 dilution of labelled streptavidin peroxidase (Dako) at room temperature for 30 min. The antibody was localised with 3,3'-diaminobenzidine tetrahydrochloride and 0.065% sodium azide was used to block endogenous peroxidase.

We stained both the deep periphery of the tumour and adjacent tumour-free tissue. A distinct nuclear immunoreaction for p53 was judged positive. In the positive cells the nuclear staining pattern was diffuse with little variation. When 10% of the cancer cells showed a positive nuclear staining, a positive staining was defined (Kakeji *et al.*, 1993).

*RB and MIB-1 staining*

Paraffin was removed in xylene and the sections then exposed to graded concentrations of alcohol. Trypsin (Difco Laboratories, USA) (0.1%) treatment was done and the slides were placed in a thermoresistant beaker filled with 0.1 M PBS (pH 7.4) and were autoclaved at 121°C. Then the sections were cooled down to room temperature for about 20 min and rinsed in PBS. The tissue sections were then covered with a monoclonal antibody 3H9 for RB protein (Medical & Biological Laboratories, Japan) (Yonemura *et al.*, 1993) or a monoclonal antibody MIB-1 for Ki-67 antigen (Immunotech, France) (Cattoretti *et al.*, 1992), with a 1:40 dilution or 1:100 dilution of antibody respectively, for 60 min at room temperature, then with biotinylated rabbit anti-mouse IgG (1:600 for 30 min), and finally with the labelled streptavidin peroxidase. Peroxidase labelling was developed with 3,3'-diaminobenzidine and sodium azide, and the sections were counterstained with haematoxylin. All stained nuclei were scored as positive for MIB-1. The RB and MIB-1 labelling indices were determined by observing 1000 nuclei in areas of the section with the highest labelling frequency and the percentage of labelled nuclei was used for analysis.

*Statistical analysis*

The BMDP Statistical Package program (BMDP; Los Angeles, CA, USA) for the IBM (Armonk, NY, USA) 4381 mainframe computer was used for all analyses (Dixon, 1988). The BMDP P4F and P3S programs were used for the chi-square test and the Mann–Whitney test to compare data on patients with and without cytokeratin-positive cells in bone marrow. The level of significance was $P < 0.05$.

### Results

*Clinicopathological factors*

Bone marrow aspirates of 46 patients with gastric cancer were examined using the monoclonal antibody CK2 directed against the cytokeratin polypeptide no. 18 that is exclusively expressed in all simple epithelia and in 100% of transformed cells derived thereof. Fifteen of 46 (32.6%) aspirates from patients comprising all tumour advances were positive for epithelial cells (Figure 1). The alkaline phosphatase-stained

**Figure 1** Microphotograph of micrometastases in the bone marrow. High-power photomicrograph of bone marrow specimens for those with one cytokeratin-positive cell (**a**) or a small cluster of cells (**b**) (original magnification × 1000).

cells in cytocentrifuge preparations appeared as red single or clustered cells, and were present at a frequency of $10^4-10^5$ nucleated cells. These cells were de-stained and confirmed as cancer cells by Papanicolaou staining. The positive findings did not depend on sex, age or location of the tumour. In cases of cytokeratin-positive cells, undifferentiated tissue type was more frequent and the depth of invasion was more prominent. Lymphatic and vascular involvement and metastasis to lymph nodes, peritoneal dissemination and liver metastasis showed no relation with the presence of micrometastasis in the bone marrow (Table I).

*Relation between the presence of micrometastasis and p53 staining*

We determined the relation between the detection of cytokeratin-positive cells in the bone marrow and abnormal p53 staining in the primary lesion. The positive rate of p53 was 25.8% (8/31) for cytokeratin-negative patients and 26.6% (4/15) for cytokeratin-positive ones, with no significant difference (Table II).

*Relation between the presence of micrometastasis and the RB staining*

The labelling index of RB staining of primary lesion was determined between the cytokeratin-negative and -positive groups. RB labelling index was 56.5 ± 25.6% for the cytokeratin-negative group and 66.9 ± 20.5% for the cytokeratin-positive group, with no significant difference (Table III).

*Relation between the presence of micrometastasis and the MIB-1 staining*

The labelling index of MIB-1 staining of primary lesion was determined between the cytokeratin-negative and -positive groups. MIB-1 staining was 60.3 ± 20.9% for the cytokeratin-negative group and 58.9 ± 20.5% for the cytokeratin-positive group, with no significant difference (Table III).

**Table I** Clinicopathological characteristics of patients with gastric cancer with and without cytokeratin-positive cells in the bone marrow

| *Variable* | *Cytokeratin-negative cases* (n=31) | *Cytokeratin-positive cases* (n=15) | P-*value* |
|---|---|---|---|
| Sex | | | |
| Male | 19 | 10 | NS |
| Female | 12 | 5 | |
| Age (years) | 61.0 ± 13.1[a] | 58.3 ± 12.7[a] | NS |
| Tumour maximal diameter (cm) | 5.60 ± 3.84[a] | 7.50 ± 3.82 | NS |
| Histology | | | |
| Differentiated | 18 | 3 | $P<0.05$ |
| Undifferentiated | 13 | 12 | |
| Depth of penetration | | | |
| Mucosa | 7 | 2 | $P<0.05$ |
| Submucosa | 7 | 0 | |
| Muscularis propria | 4 | 1 | |
| Subserosa | 6 | 6 | |
| Serosa | 6 | 5 | |
| Invasion into adjacent organs | 1 | 1 | |
| Lymph node metastasis | | | |
| Negative | 15 | 8 | NS |
| Positive | 16 | 7 | |
| Peritoneal dissemination | | | |
| Negative | 30 | 12 | NS |
| Positive | 1 | 3 | |
| Liver metastasis | | | |
| Negative | 31 | 14 | NS |
| Positive | 0 | 1 | |

[a]mean ± standard deviation; NS, not significant.

**Table II** Rate of p53 overexpression in gastric cancer with or without cytokeratin-positive cells in the bone marrow

| *Factor* | *Without cytokeratin-positive cells* (n=31) | *With cytokeratin-positive cells* (n=15) | P-*value* |
|---|---|---|---|
| p53 expression | | | |
| Negative | 23 | 11 | NS |
| Positive | 8 | 4 | |

NS, not significant.

**Table III** Biological character evaluated by RB and MIB-1 labelling in gastric cancer with or without cytokeratin-positive cells in the bone marrow

| *Factor* | *Without cytokeratin-positive cells* (n=31) | *With cytokeratin-positive cells* (n=15) | P-*value* |
|---|---|---|---|
| RB labelling (%) | 56.5 ± 25.6[a] | 66.9 ± 31.4 | NS |
| MIB-1 labelling (%) | 60.3 ± 20.9[a] | 58.9 ± 20.5 | NS |

[a]Mean ± standard deviation. NS, not significant.

### Discussion

Even after a curative resection there can be recurrences in patients with advanced or even early gastric cancer (Ichiyoshi *et al.*, 1990; Maehara *et al.*, 1992). A highly sensitive method is needed to predict metastatic potential and clinical outcome and to design pertinent treatments. Gastric cancer markers may provide prognostic information independent of and complementary to conventional parameters, including growth potential, oncogenes, tumour-suppressor genes and DNA flow cytometry, as well as other growth factors (Korenaga *et al.*, 1990; Mitzutani *et al.*, 1993; Joypaul *et al.*, 1994). The common characteristic of these prognostic factors is that they correlate a property of the primary tumour with the subsequent outcome.

The method we have described here relates to an aspect of the actual behaviour of the tumour, microscopic dissemination of cancer cells in the bone marrow. Cytokeratin expression is conserved in nearly all normal epithelial cells as well as in primary and metastatic carcinoma cells (Debus *et al.*, 1984) and is absent in haematopoietic and lymphatic cells. As cytokeratin-positive cells were not detected in bone marrow, they are suitable to detect micrometastases present in the marrow (Schlimok and Riethmüller, 1990*a*). There are reports of micrometastasis in the bone marrow of patients with colorectal or breast cancer (Mansi *et al.*, 1987; Schlimok and Riethmüller, 1990*a*; Lindemann *et al.*, 1992; Pantel *et al.*, 1993*a*). The evidence of micrometastasis meant an early relapse and the clinical outcome for these patients could be predicted (Schlimok *et al.*, 1990*b*; Cote *et al.*, 1991; Lindemann *et al.*, 1992;). We also found cytokeratin-positive cells in lymph nodes in 22% of our patients with node-negative early gastric cancer and who died with a recurrence (Maehara *et al.*, 1995).

In the present report, clinicopathological features and the malignant potential of primary tumour were examined for patients with epithelial cells in their marrow at the time of surgery for the primary tumour. The presence of micrometastatic cells at this time correlated with tissue differentiation and the depth of invasion. Micrometastasis was noted in two patients with mucosal gastric cancer, thus seeding of cancer cells can occur even in the early stage of the cancer. Schlimok & Riethmüller (1990*a*) found the cytokeratin-positive rate to be 12.5% for node-negative gastric cancer, but in our patients the rate was 8/23 (34.8%). We examined the biological nature of cancer cells in the primary lesion by determining the expression of p53 and RB proteins and

MIB-1 staining. The p53 and RB abnormalities are reported to be related to aggressive behaviour of cancer cells for serosal invasion, lymph node metastasis, peritoneal dissemination and liver metastasis of gastric cancer, and the prognosis was poor (Yonemura *et al.*, 1993; Joypaul *et al.*, 1994). MIB-1 reacts with a nuclear non-histone protein (Ki-67 antigen) in all active parts of the cell cycle and the level of labelling index relates to lymphatic and vascular progression (Cattoretti *et al.*, 1992). As similar distributions of markers of tumour malignancy and proliferation were found on the primary lesion, irrespective of the presence of cytokeratin-positive cells in bone marrow, characteristics of the metastasis to bone marrow and the outgrowth of tumour cells into visible metastases need to be clarified.

Overt bone or skeleton metastases are rare in patients with gastric cancer, however, bone marrow is distinctly more often involved than expected from the clinical findings (Koga *et al.*, 1987; Lindemann *et al.*, 1992). The apparent discrepancy between clinically rare bone metastases and the marrow micrometastases frequently detected by immunocytochemistry may be explained by a reduced proliferative behaviour of the cells and often invoked state of dormancy (Schlimok *et al.*, 1990*b*; Cote *et al.*, 1991). The capacity of the tumour cells to proliferate in the bone marrow and to manifest metastasis depends on the microenvironment. The presence of disseminated cells in bone marrow could also indicate that cancer cells have reached the peritoneum, liver or lung (Lindemann *et al.*, 1992). Therefore, these patients may have complications arising from peritoneal dissemination or liver metastasis with no manifest metastasis in the bone marrow (Maehara *et al.*, 1991; Moriguchi *et al.*, 1992).

Cytokeratin-positive cells in the bone marrow of gastric cancer patients may serve as valid indicators of the intrinsic metastatic activity of an individual tumour. Patients presenting with disseminated cytokeratin-positive cells at the time of primary surgery have to be closely followed and the possible presence of distant metastasis should always be a concern for attending physicians.

**Acknowledgements**
This work was supported by Grant-in-Aid for Scientific Research on Priority Areas (regarding Cancer Research) (06282115) from the Ministry of Education, Science and Culture in Japan. We thank M Ohara for comments and H Baba and J Tsuchihashi for technical assistance.

### References

CATTORETTI G, BECKER MHG, KEY G, DUCHROW M, SCHLÜTER C, GALLE J AND GERDES J. (1992). Monoclonal antibodies against recombinant parts of the Ki-67 antigen (MIB1 and MIB3) detect proliferating cells in microwave-processed formalin-fixed paraffin sections. *J. Pathol.*, **168**, 357-363.

COTE RJ, ROSEN PP, LESSER ML, OLD LJ AND OSBORNE MP. (1991). Prediction of early relapse in patients with operable breast cancer by detection of occult bone marrow micrometastases. *J. Clin. Oncol.*, **9**, 1749-1756.

DEBUS E, MOLL R, FRANKE WW, WEBER K AND OSBORN M. (1984). Immunohistochemical distinction of human carcinomas by cytokeratin typing with monoclonal antibodies. *Am. J. Pathol.*, **114**, 121-130.

DIXON WJ. (ed). (1988). *BMDP Statistical Software*. University of California Press: Berkeley.

GUESDON J-L, TERNYNCK T AND AVRAMEAS S. (1979). The use of avidin-biotin interaction in immunoenzymatic techniques. *J. Histochem. Cytochem.*, **27**, 1131-1139.

ICHIYOSHI Y, TODA T, MINAMISONO Y, NAGASAKI S, YAKEISHI Y AND SUGIMACHI K. (1990). Recurrence in early gastric cancer. *Surgery*, **107**, 489-495.

JAPANESE RESEARCH SOCIETY FOR GASTRIC CANCER. (1981*a*). The general rules for the gastric cancer study in surgery and pathology. Part I. Clinical Classification. *Jpn. J. Surg.*, **11**, 127-139.

JAPANESE RESEARCH SOCIETY FOR GASTRIC CANCER. (1981*b*). The general rules for the gastric cancer study in surgery and pathology. Part II. Histological classificatioin of gastric cancer. *Jpn. J. Surg.*, **11**, 140-145.

JAPANESE RESEARCH SOCIETY FOR GASTRIC CANCER. (1993). *The General Rules for Gastric Cancer Study*. 12th edn. (in Japanese). Kanehara and Company: Tokyo.

JOYPAUL BV, HOPWOOD D, NEWMAN EL, QURESHI S, GRANT A, OGSTON SA, LANE DP AND CUSCHIERI A. (1994). The prognostic significance of the accumulation of p53 tumour-suppressor gene protein in gastric adenocarcinoma. *Br. J. Cancer*, **69**, 943-946.

KAKEJI Y, KORENAGA D, TSUJITANI S, BABA H, ANAI H, MAEHARA Y AND SUGIMACHI K. (1993). Gastric cancer with p53 overexpression has high potential for metastasising to lymph nodes. *Br. J. Cancer*, **67**, 589-593.

KOGA S, TAKEBAYASHI M, KAIBARA N, NISHIDOI H, KIMURA O, KAWASUMI H AND MAKINO M. (1987). Pathological characteristics of gastric cancer that develop hematogenous recurrence, with special reference to the site of recurrence. *J. Surg. Oncol.*, **36**, 239-242.

KORENAGA D, SAITO A, BABA H, WATANABE A, OKAMURA T, MAEHARA Y AND SUGIMACHI K. (1990). Cytophotometrically determined DNA content, mitotic activity, and lymph node metastasis in clinical gastric cancer. *Surgery*, **107**, 262-267.

LAUWERS GY, WAHL SJ, MELAMED J AND ROJAS-CORONA RR. (1993). p53 expression in precancerous gastric lesions: An immunohistochemical study of PAb 1801 monoclonal antibody on adenomatous and hyperplastic gastric polyps. *Am. J. Gastroenterol.*, **88**, 1916-1919.

LINDEMANN F, SCHLIMOK G, DIRSCHEDL P, WITTE J AND RIETHMÜLLER G. (1992). Prognostic significance of micrometastatic tumour cells in bone marrow of colorectal cancer patients. *Lancet*, **340**, 685-689.

MAEHARA Y, MORIGUCHI S, KAKEJI Y, KOHNOE S, KORENAGA D, HARAGUCHI M AND SUGIMACHI K. (1991). Pertinent risk factors and gastric carcinoma with synchronous peritoneal dissemination or liver metastasis. *Surgery*, **110**, 820-823.

MAEHARA Y, OKUYAMA T, MORIGUCHI S, ORITA H, KUSUMOTO H, KORENAGA D AND SUGIMACHI K. (1992). Prophylactic lymph node dissection in patients with advanced gastric cancer promotes increased survival time. *Cancer*, **70**, 392-395.

MAEHARA Y, BABA H, OHNO S AND SUGIMACHI K. (1995). Cytokeratin staining reveals micrometastasis in lymph nodes of early gastric cancer. *Surgery*, **117**, 480.

MANSI JL, BERGER U, EASTON D, MCDONNELL T, REDDING WH, GAZET J-C, MCKINNA A, POWLES TJ AND COOMBES RC. (1987). Micrometastases in bone marrow in patients with primary breast cancer: evaluation as an early predictor of bone metastases. *Br. Med. J.*, **295**, 1093-1096.

MIZUTANI T, ONDA M, TOKUNAGA A, YAMANAKA N AND SUGISAKI Y. (1993). Relationship of C-*erb*B-2 protein expression and gene amplification to invasion and metastasis in human gastric cancer. *Cancer*, **72**, 2083-2088.

MORIGUCHI S, MAEHARA Y, KORENAGA D, SUGIMACHI K AND NOSE Y. (1992). Risk factors which predict pattern of recurrence after curative surgery for patients with advanced gastric cancer. *Surg. Oncol.*, **1**, 341-346.

OBERNEDER R, RIESENBERG R, KRIEGMAIR M, BITZER U, KLAMMERT R, SCHNEEDE P, HOFSTETTER A, RIETHMÜLLER G AND PANTEL K. (1994). Immunocytochemical detection and phenotypic characterization of micrometastatic tumour cells in bone marrow of patients with prostate cancer. *Urol. Res.*, **22**, 3-8.

PANTEL K, IZBICKI JR, ANGSTWURM M, BRAUN S, PASSLICK B, KARG O, THETTER O AND RIETHMÜLLER G. (1993*a*). Immunocytological detection of bone marrow micrometastasis in operable non-small cell lung cancer. *Cancer Res.*, **53**, 1027-1031.

PANTEL K, BRAUN S, SCHLIMOK G AND RIETHMÜLLER G. (1993*b*). Micrometastatic tumour cells in bone marrow in colorectal cancer. *Lancet*, **341**, 501.

RICHARD JC, ROSEN PP, LESSER ML, OLD LJ AND OSBORNE MP. (1991). Prediction of early relapse in patients with operable breast cancer by detection of occult bone marrow micrometastases. *J. Clin. Oncol.*, **9**, 1749-1756.

SCHLIMOK G AND RIETHMÜLLER G. (1990*a*). Detection, characterization and tumorigenicity of disseminated tumor cells in human bone marrow. *Semin. Cancer Biol.*, **1**, 207–215.

SCHLIMOK G, FUNKE I, BOCK B, SCHWEIBERER B, WITTE J AND RIETHMÜLLER G. (1990*b*). Epithelial tumour cells in bone marrow of patients with colorectal cancer: Immunocytochemical detection, phenotypic characterization, and prognostic significance. *J. Clin. Oncol.*, **8**, 831–837.

YONEMURA Y, NINOMIYA I, TSUGAWA K, SUGIYAMA K, FUJIMURA T, HIRONO Y, TAKAMURA H, MIYAZAKI I, ENDOU Y, TANAKA M AND SASAKI T. (1993). Correlation between altered expression of retinoblastoma protein and clinical outcome in patients with gastric cancer. *Int. J. Oncol.*, **3**, 71–75.